# Endometriosis: Epidemiology, Risk Factors, Molecular Mechanisms, Diagnosis, and Management

**DOI:** 10.1002/mco2.70852

**Published:** 2026-07-08

**Authors:** Kun Wang, Dongyun He, Yang Wang, Xiaojun Liu, Li Liu

**Affiliations:** ^1^ Department of Obstetrics China‐Japan Union Hospital of Jilin University Changchun Jilin China; ^2^ Reproductive Medicine Center China‐Japan Union Hospital of Jilin University Changchun Jilin China; ^3^ Department of dermatology Affiliated Hospital of Changchun University of Chinese Medicine Changchun Jilin China

**Keywords:** diagnosis, endometriosis, epidemiology, management, molecular mechanisms

## Abstract

Endometriosis is a chronic estrogen‐dependent gynecological disorder characterized by the ectopic growth of endometrial‐like tissue outside the uterine cavity, affecting approximately 10% of women of reproductive age worldwide. It is a leading cause of chronic pelvic pain, dysmenorrhea, and infertility, imposing a substantial burden on individual health and healthcare systems. Despite decades of research, the pathogenesis of endometriosis remains incompletely elucidated, and clinical management is often limited by delayed diagnosis and suboptimal treatment efficacy. This review provides a comprehensive and updated overview of endometriosis, covering its updated epidemiology, established and emerging risk factors, and key molecular mechanisms including inflammation, epigenetic regulation, hormonal imbalance, oxidative stress, and abnormal cell invasion and proliferation. We further summarize current diagnostic approaches, including imaging modalities, laparoscopy, and biomarkers, as well as clinical management strategies such as hormonal therapy, surgical intervention, and emerging targeted treatments. Finally, we discuss existing diagnostic and therapeutic challenges, unresolved questions, and future directions toward precision medicine and noninvasive management. This review aims to integrate recent advances and provide an updated framework for understanding and improving the clinical care of endometriosis. This review also highlights actionable translational directions for noninvasive early diagnosis, precision targeted therapy, and postoperative recurrence prevention, providing a key theoretical framework for translating basic research findings into clinical practice.

## Graphical abstract

*Global epidemiology, integrated determinants, and disease burden of endometriosis*. This circular schematic comprehensively illustrates the global prevalence and demographic variation of endometriosis, its multifactorial integrated determinants, the multifaceted disease burden and economic impact, and recent epidemiological trends, providing a holistic overview of the epidemiological landscape of endometriosis.

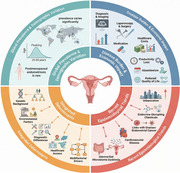

## 1 Introduction

Endometriosis was first formally described by von Rokitansky in 1860, but its clinical recognition and systematic research did not gain traction until the 20th century [1]. Initially dismissed as a rare pathological curiosity, endometriosis is now recognized as one of the most prevalent gynecological disorders, with a global prevalence of approximately 6–10% in women of reproductive age [2, 3]. The clinical manifestations of endometriosis are highly heterogeneous, ranging from asymptomatic cases to severe chronic pelvic pain (CPP), dysmenorrhea, dyspareunia, and infertility [4]. Notably, up to 50% of women with endometriosis experience infertility, and 70–80% suffer from chronic pain that significantly impairs quality of life [5]. Beyond reproductive health, endometriosis is associated with long‐term comorbidities including ovarian cancer, autoimmune diseases, and mental health disorders such as anxiety and depression [6, 7], highlighting the need for comprehensive management strategies. Despite its high prevalence and profound clinical impact, endometriosis remains underdiagnosed globally, with an average diagnostic delay of 7–10 years from symptom onset, exacerbating disease progression and patient suffering. This diagnostic gap underscores the urgent need for improved early screening tools, enhanced clinical awareness, and targeted therapeutic interventions to address this unmet global health burden.

Over the past decade, significant progress has been made in understanding the pathophysiology of endometriosis. Key advances include the identification of genetic susceptibility loci, the recognition of the role of the microbiome in disease development, and the discovery of novel molecular pathways driving ectopic tissue growth [8, 9, 10]. However, several critical gaps remain: (1) *gaps in pathogenesis and pathophysiology*: unclear mechanisms of ectopic endometrial dissemination and implantation; ambiguous role of the microbiome; unresolved core contradictions in hormonal regulation. (2) *Diagnosis‐related gaps*: Lack of noninvasive biomarkers with high sensitivity and specificity; scarcity of early diagnostic technologies; absence of diagnostic classification based on disease heterogeneity. (3) *Treatment‐related gaps*: lack of disease‐modifying therapies; absence of personalized treatment strategies; blank in the prevention and treatment of long‐term complications. (4) *Gaps in disease heterogeneity and classification*: unidentified molecular subtypes; unclear gene–environment interactions. These unmet challenges collectively hinder the development of precision medicine approaches, perpetuating the global burden of endometriosis and underscoring the urgent need for this comprehensive review to synthesize current advances and outline future priorities.

Given the high prevalence, clinical impact, and unresolved questions surrounding endometriosis, this review synthesizes updated epidemiological data, novel molecular insights, and state‐of‐the‐art clinical advances, with a special focus on the gut microbiota‐endometriosis axis as a novel mechanistic and therapeutic target. This review is structured sequentially to cover epidemiology, risk factors, molecular mechanisms, diagnosis, clinical management, preclinical models, clinical trials, and future perspectives to ensure logical flow and readability for clinicians and researchers alike.

## 2 Epidemiology of Endometriosis

Section 2 systematically presents the global epidemiological profile of endometriosis, including regional and demographic variations in prevalence, the individual and socioeconomic disease burden, and recent emerging trends linked to environmental factors and microbiome dysregulation (Table 1).

**TABLE 1:** Global epidemiology and major risk factors of endometriosis.

Category	Key indicators	Summary data
Global prevalence	General reproductive‐aged women	6–10% [13]
	Women with infertility or chronic pelvic pain	30–50% [13]
Age distribution	Peak onset age	25–34 years [12]
Economic burden	Mean annual cost per patient	Int $16,970–20,898 [18]
Strong risk factors	First‐degree family history	7–10‐fold increased risk [28]
	EDCs (BPA, phthalates) exposure	2–3‐fold increased risk [37, 38]
	Low BMI and insufficient physical activity	1.5–2‐fold increased risk [58, 59]

Abbreviations: BMI, body mass index; EDCs, endocrine‐disrupting chemicals; BPA, bisphenol A

### 2.1 Global Prevalence and Demographic Variations

Endometriosis is a globally distributed disorder, but its prevalence varies significantly across regions, populations, and diagnostic methods [11, 12]. The overall prevalence in women of reproductive age (15–49 years) is estimated at 6–10%, but this figure rises to 30–50% in women with infertility or CPP [13]. Regional variations are notable: for example, the prevalence is approximately 10% in North America and Europe, 8–12% in East Asia, and 5–8% in Africa and South America [14, 15]. These differences may reflect variations in diagnostic practices, genetic background, environmental factors, and access to healthcare. Age is a critical demographic factor for endometriosis, which typically presents in women aged 25–34 years (peaking in the third decade of life) but affects reproductive‐aged women more broadly, yet the molecular mechanisms driving age‐related differences in disease onset, severity, and subtype distribution remain poorly understood [12]. Postmenopausal endometriosis is rare, occurring in less than 1% of cases, and is often associated with exogenous estrogen exposure or hormone‐secreting tumors [16]. Notably, true population‐based prevalence estimates remain challenging to obtain due to the reliance on surgical diagnosis, which excludes asymptomatic and undiagnosed cases, leading to potential underestimation of the global disease burden. Additionally, emerging data suggest rising prevalence trends in urbanized regions, potentially linked to lifestyle changes and increased exposure to endocrine‐disrupting chemicals (EDCs).

### 2.2 Disease Burden and Economic Impact

Endometriosis imposes a substantial individual and societal burden. The direct healthcare costs include diagnostic tests (e.g., imaging, laparoscopy), medications, and surgical interventions, while indirect costs stem from lost productivity, absenteeism, and reduced quality of life [17]. According to an analysis of 14 published studies, endometriosis confers a considerable socioeconomic burden driven by direct medical expenditures and reduced workplace productivity. Cross‐country comparisons reveal substantial variations in the mean annual cost per patient, from EUR 8768 in Sweden to Int $16,970–20,898 in Australia. In the United States, nonsurgical interventions exhibited superior cost‐effectiveness relative to surgical approaches, with an incremental cost of $100,000 per QALY gained. In the United Kingdom, the LNG‐IUS was associated with the highest treatment cost at £650.94. In China, expenditures associated with distinct therapeutic strategies reached up to $10,728, accompanied by divergent ICER values across interventions [18, 19]. These regional disparities reflect differences in healthcare systems, treatment accessibility, and diagnostic efficiency‐with low‐ and middle‐income countries often facing underreported burdens due to limited specialized care and diagnostic resources. Globally, the burden is projected to increase as awareness and diagnosis rates improve [17, 20]. The impact on quality of life is comparable to that of chronic conditions such as diabetes, rheumatoid arthritis, and Crohn's disease, with women reporting significant impairments in physical, emotional, and social functioning. CPP, infertility‐related distress, and repeated treatment cycles further exacerbate psychological burden, leading to higher rates of anxiety, depression, and healthcare‐seeking fatigue. Additionally, the invisible nature of many symptoms often results in unrecognized workplace challenges, including reduced work capacity and career advancement limitations, which amplify long‐term economic impacts for both individuals and societies.

### 2.3 Recent Epidemiological Trends

Recent epidemiological studies have highlighted several emerging trends. First, the prevalence of endometriosis appears to be increasing, particularly in urban areas, which may be attributed to environmental factors such as exposure to EDCs [21, 22]. Urbanization‐related lifestyle shifts‐including delayed childbearing, reduced parity, and increased stress‐may further synergize with EDC exposure to amplify disease risk, as these factors disrupt hormonal balance and immune homeostasis. Second, there is growing recognition of the association between endometriosis and other chronic diseases, including ovarian cancer, endometrial cancer, and cardiovascular disease [23, 24]. For example, women with endometriosis have a twofold to threefold increased risk of epithelial ovarian cancer, particularly clear cell and endometrioid subtypes [25]. This comorbidity pattern suggests shared pathogenic mechanisms, such as chronic inflammation, oxidative stress, and epigenetic dysregulation, which may drive the development of multiple chronic conditions over time. Third, epidemiological data increasingly support the role of the microbiome in endometriosis: recent studies have shown that uterine and gut microbiota dysbiosis is associated with an increased risk of endometriosis [26, 27]. Notably, gut microbiota alterations may modulate estrogen metabolism and systemic inflammation, while uterine dysbiosis disrupts the endometrial barrier function, creating a permissive environment for ectopic tissue implantation. These trends underscore the need for a holistic approach to understanding and managing endometriosis.

**FIGURE 1:**
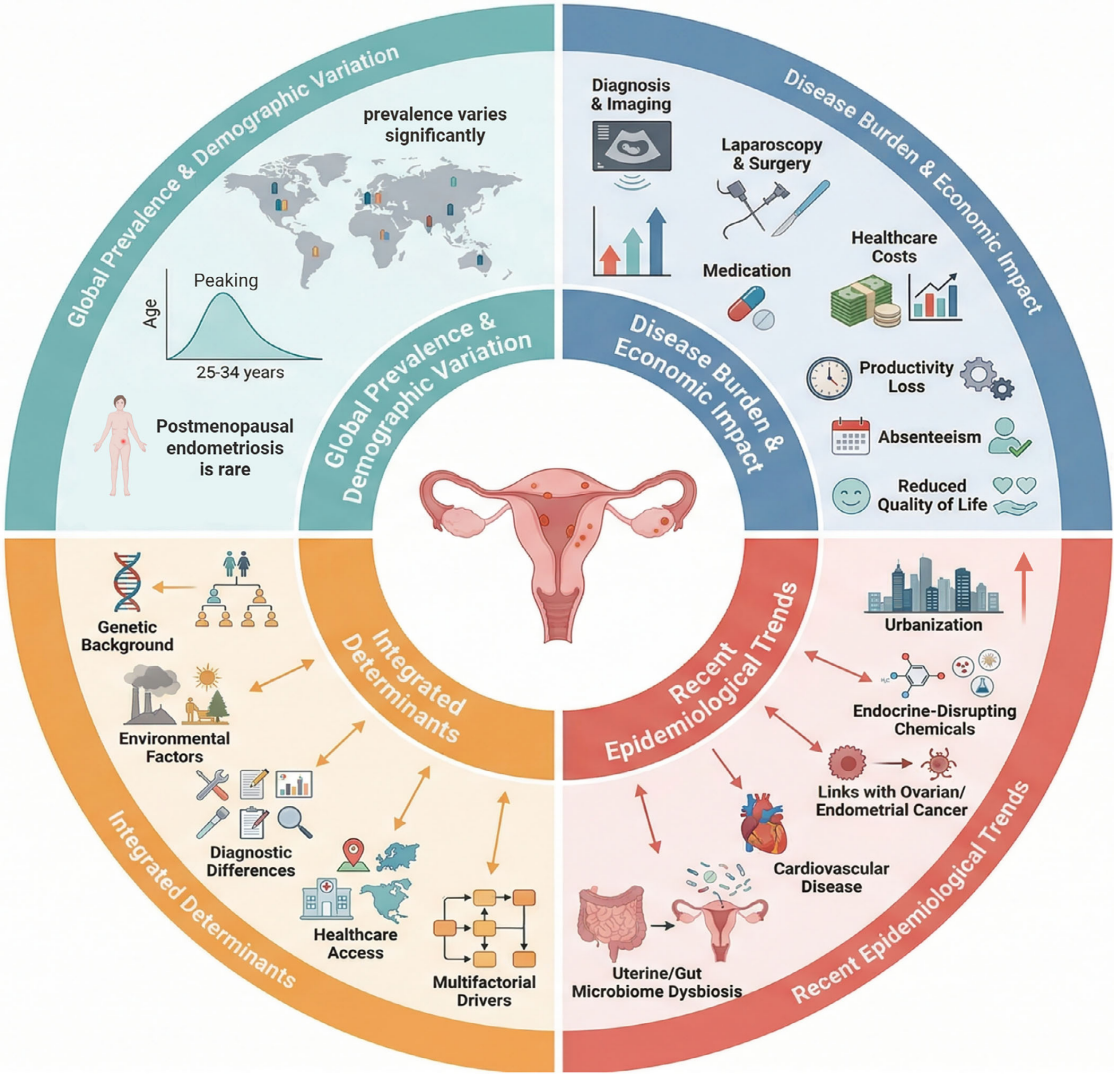
Global epidemiology, integrated determinants, and disease burden of endometriosis. This circular schematic provides a holistic and systematic overview of the global epidemiological landscape of endometriosis, covering multidimensional components including prevalence distribution, demographic characteristics, disease burden, and emerging trends. It displays regional variations in prevalence across North America, Europe, East Asia, Africa, and South America, with a peak incidence among women aged 25–34 years. The figure integrates major determinants such as genetic susceptibility, endocrine‐disrupting chemical exposure, dietary habits, BMI, physical activity, and hormonal imbalance. It also quantifies the comprehensive disease burden, including chronic pelvic pain, infertility, psychological disorders, elevated ovarian cancer risk, and substantial economic costs from direct medical expenses and lost productivity. Recent epidemiological trends, including rising urban prevalence, comorbidity with cardiovascular diseases, and the involvement of gut microbiota dysbiosis, are also highlighted. This figure was created with BioRender.com.

Endometriosis is a highly prevalent disorder with significant global variation in prevalence, driven by demographic, genetic, environmental, and healthcare factors. The disease imposes a substantial economic and quality‐of‐life burden, with emerging links to other chronic conditions. Recent epidemiological trends highlight the role of environmental factors and the microbiome, emphasizing the need for integrated research to address the complex determinants of endometriosis (Figure 1).

## 3 Risk Factors for Endometriosis

Section 3 comprehensively summarizes the multifactorial risk profile of endometriosis, covering genetic predisposition, environmental exposures, and modifiable lifestyle factors, with updated evidence on the gut microbiome as an emerging determinant.

### 3.1 Genetic Factors

#### 3.1.1 Family History and Heritability

Family history is one of the strongest risk factors for endometriosis: women with a first‐degree relative with endometriosis have a 7–10‐fold increased risk of developing the disease [28]. Twin studies have confirmed the heritability of endometriosis, with heritability estimates ranging from 30 to 50% [29, 30]. These findings suggest that genetic factors play a significant role in disease susceptibility, but environmental factors also contribute to disease expression.

#### 3.1.2 Genome‐Wide Association Studies

Over the past decade, genome‐wide association studies (GWAS) have identified numerous genetic loci associated with endometriosis [31, 32]. The first large‐scale GWAS identified two loci on chromosomes 10q26 and 7p15.2 [31]. Subsequent GWAS, including meta‐analyses of over 150,000 cases and controls, have expanded the list to more than 40 susceptibility loci [33]. These loci are involved in a range of biological processes, including estrogen signaling (e.g., ESR1, PGR), inflammation (e.g., interleukin [IL]‐6, tumor necrosis factor [TNF]), and cell adhesion and invasion (e.g., CDKN2A, CDKN2B) [34, 35]. Notably, many of these loci are shared with other inflammatory and reproductive disorders, such as rheumatoid arthritis and polycystic ovary syndrome, suggesting common pathogenic pathways [36]. Functional annotation of these loci has highlighted key roles in immune cell regulation, steroid hormone biosynthesis, and tissue remodeling, providing mechanistic insights into how genetic variants predispose to endometriosis. Integration of multiomics data with GWAS results is further unraveling causal genes and regulatory networks underlying disease risk.

### 3.2 Environmental Factors

#### 3.2.1 Endocrine‐Disrupting Chemicals

Exposure to EDCs is a well‐established environmental risk factor for endometriosis. EDCs are compounds that interfere with the endocrine system, including bisphenol A (BPA), phthalates, polychlorinated biphenyls, and dioxins [37, 38]. Animal studies have shown that prenatal or postnatal exposure to EDCs increases the risk of endometriosis‐like lesions [39]. Human studies have consistently linked EDC exposure to endometriosis: for example, women with higher urinary BPA levels have a twofold to threefold increased risk of endometriosis [40, 41, 42], and exposure to phthalates is associated with an increased risk of severe endometriosis [43, 44]. The mechanisms by which EDCs promote endometriosis include modulation of estrogen receptor activity, induction of oxidative stress, and disruption of the microbiome [45].

#### 3.2.2 Other Environmental Factors

Other environmental factors associated with endometriosis include cigarette smoking, alcohol consumption, and air pollution [46]. Cigarette smoking has been shown to have a dual effect: heavy smoking is associated with a reduced risk of endometriosis, possibly due to antiestrogenic effects, but light smoking may increase the risk of severe endometriosis [47]. Alcohol consumption is associated with a 1.5–2‐fold increased risk, likely due to increased estrogen levels and oxidative stress [48]. Air pollution, particularly exposure to particulate matter (PM2.5) and nitrogen dioxide, has also been linked to endometriosis, with mechanisms involving inflammation and epigenetic modifications [49].

### 3.3 Lifestyle Factors

#### 3.3.1 Diet and Nutrition

Dietary factors play a role in endometriosis risk [50]. A high intake of red meat, saturated fats, and refined carbohydrates is associated with an increased risk, while a diet rich in fruits, vegetables, whole grains, and omega‐3 fatty acids is associated with a reduced risk [51]. For example, omega‐3 fatty acids have anti‐inflammatory properties that may mitigate endometriosis development [52]. Vitamin D deficiency is also a risk factor: women with vitamin D levels have a twofold increased risk of endometriosis [53, 54]. Notably, dietary patterns such as the Mediterranean diet‐high in plant‐based foods, fish, and olive oil‐have been specifically linked to lower risk, likely due to synergistic anti‐inflammatory and antioxidant effects. Additionally, excessive sugar intake may exacerbate risk by promoting oxidative stress and insulin resistance, which disrupt hormonal balance.

#### 3.3.2 Body Mass Index and Physical Activity

Body mass index (BMI) is inversely associated with endometriosis risk: women with a BMI/m^2^ have a 1.5–2‐fold increased risk, while obesity (BMI ≥ 30 kg/m^2^) is associated with a reduced risk [55, 56]. This inverse relationship may be explained by differences in estrogen levels (obese women have higher levels of endogenous estrogen, but adipose tissue may also secrete anti‐inflammatory cytokines) [57]. However, this association is controversial in severe endometriosis subtypes, where obesity may not confer protection and could even worsen outcomes by amplifying chronic inflammation. Physical activity is associated with a reduced risk of endometriosis: women who engage in ≥3 h of moderate‐to‐vigorous physical activity per week have a 30–40% lower risk [58, 59], likely due to reduced estrogen levels, improved insulin sensitivity, and anti‐inflammatory effects. Even low‐to‐moderate activity, such as brisk walking or yoga, may offer protective benefits, making physical activity an accessible preventive strategy for diverse populations.

**FIGURE 2:**
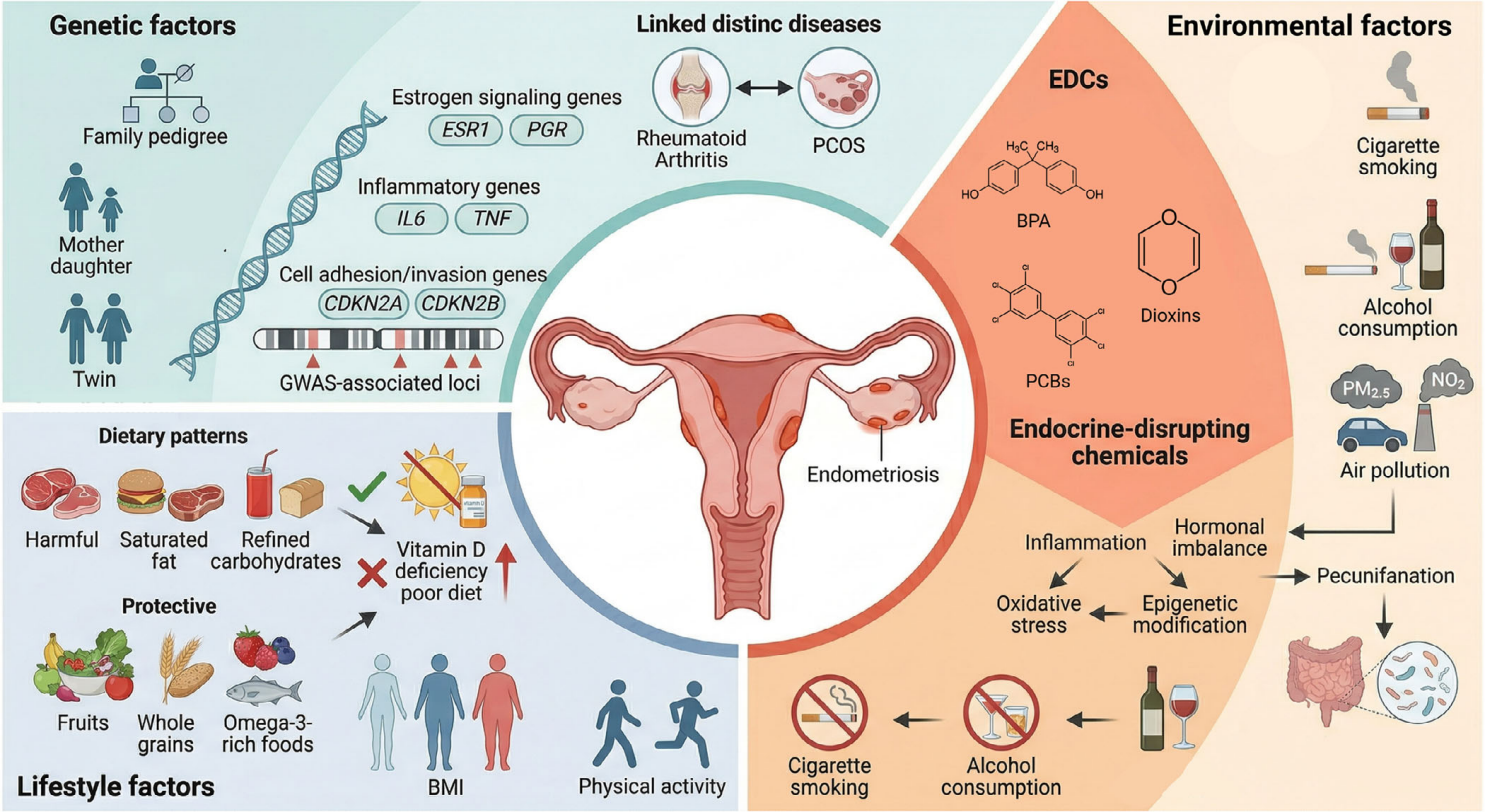
Multifactorial pathogenesis network of endometriosis. This schematic illustrates the integrated and interactive pathogenesis network of endometriosis from genetic, environmental, hormonal, and lifestyle dimensions. It demonstrates how genetic predisposition and family history contribute to disease susceptibility, while environmental factors such as endocrine‐disrupting chemicals, air pollution, and smoking promote pathological progression. Hormonal imbalance, particularly excessive estrogen signaling and progesterone resistance, serves as a core driving force. Concurrently, chronic inflammation, oxidative stress, and epigenetic dysregulation including abnormal DNA methylation and noncoding RNA regulation further amplify disease development. Lifestyle factors including high‐fat diet, vitamin D deficiency, low BMI, and insufficient physical activity act as critical modulators. Together, these factors form a synergistic regulatory cascade that promotes the implantation, proliferation, and persistence of ectopic endometrial lesions. This figure was created with BioRender.com.

Endometriosis is a multifactorial disorder with genetic, environmental, and lifestyle risk factors. Genetic factors, including family history and GWAS‐identified loci, contribute to disease susceptibility. Environmental factors such as EDC exposure, cigarette smoking, and air pollution promote disease development, while lifestyle factors including diet, BMI, and physical activity modulate risk (Figure 2). Understanding these risk factors is critical for developing preventive strategies and identifying high‐risk populations for early screening.

## 4 Molecular Mechanisms of Endometriosis

Section 4 dissects the core pathogenic pathways of endometriosis, emphasizing the crosstalk and hierarchical regulatory relationships among inflammation, epigenetics, hormonal imbalance, oxidative stress, and abnormal cell behavior, rather than a simple listing of individual mechanisms.

### 4.1 Inflammation and Immune Dysregulation

#### 4.1.1 Cytokines and Chemokines

Inflammation is a central feature of endometriosis, with the ectopic endometrial tissue and surrounding microenvironment characterized by increased levels of proinflammatory cytokines and chemokines [60]. Key cytokines involved include tumor necrosis factor‐α (TNF‐α), IL‐1β, IL‐6, IL‐8, and transforming growth factor‐β [61, 62]. These cytokines promote ectopic tissue growth by stimulating cell proliferation, angiogenesis, and invasion, and by suppressing immune surveillance [63]. For example, TNF‐α induces the expression of matrix metalloproteinases (MMPs), which degrade the extracellular matrix (ECM) and facilitate cell invasion [64]. Chemokines such as CXCL12 and CCL2 recruit immune cells to the ectopic site, further amplifying the inflammatory response [65]. This cytokine–chemokine network also disrupts the peritoneal microenvironment homeostasis, inhibiting normal tissue repair and promoting fibroblast activation, which contributes to adhesion formation and disease chronicity.

#### 4.1.2 Immune Cell Dysfunction

Immune cell dysfunction is a key component of endometriosis pathogenesis [66]. The endometrium and peritoneal cavity contain a variety of immune cells, including macrophages, T cells, B cells, and natural killer (NK) cells [67]. In women with endometriosis, macrophages are present in increased numbers and exhibit a proinflammatory phenotype, secreting high levels of cytokines and growth factors [68]. T cells are also dysregulated: there is an imbalance between proinflammatory Th1 cells and anti‐inflammatory Th2 cells, with a shift toward Th2 dominance [69, 70]. NK cell function is impaired, with reduced cytotoxicity against ectopic endometrial cells, allowing them to evade immune destruction [71, 72]. Additionally, regulatory T cells are overexpressed, further suppressing antitumor‐like immune responses and promoting immune tolerance to ectopic tissue. These immune cell abnormalities contribute to the establishment and progression of endometriosis.

### 4.2 Epigenetic Regulation

#### 4.2.1 DNA Methylation

Epigenetic modifications, including DNA methylation, histone modification, and noncoding RNA (ncRNA) regulation, play a critical role in endometriosis pathogenesis [73, 74]. DNA methylation is the most well‐studied epigenetic mechanism in endometriosis: aberrant methylation of genes involved in estrogen signaling, inflammation, and cell adhesion has been observed in ectopic endometrial tissue [75, 76]. For example, hypermethylation of the estrogen receptor α (ESR1) gene reduces ESR1 expression, leading to increased estrogen sensitivity [77, 78]. Hypomethylation of the MMP9 gene increases its expression, promoting cell invasion [79]. These DNA methylation changes are thought to be induced by environmental factors such as EDCs and inflammation [75, 80]. Notably, DNA methylation patterns are often tissue‐specific and stable, making them potential diagnostic biomarkers and therapeutic targets. Epigenetic modifiers like DNA methyltransferase inhibitors can reverse aberrant methylation, offering a novel approach to disease modification.

#### 4.2.2 Histone Modification and ncRNAs

Histone modifications, including acetylation, methylation, and phosphorylation, also regulate gene expression in endometriosis [81]. For example, increased histone acetylation of the IL‐6 gene promoter enhances its transcription, contributing to inflammation [82]. NcRNAs, including microRNAs (miRNAs), long ncRNAs (lncRNAs), and circular RNAs (circRNAs), are emerging as key regulators of endometriosis [83, 84]. MiRNAs such as miR‐143, miR‐145, and miR‐200c are dysregulated in endometriosis, targeting genes involved in cell proliferation, invasion, and angiogenesis [85, 86]. LncRNAs such as HOTAIR and MALAT1 promote endometriosis by regulating gene expression through epigenetic mechanisms [87, 88]. CircRNAs, such as circRNA_0001821, act as miRNA sponges, modulating downstream target genes [89]. The crosstalk between histone modifications and ncRNAs further amplifies epigenetic dysregulation, forming complex regulatory networks that drive ectopic tissue survival and progression. These interactive mechanisms are increasingly targeted in preclinical studies.

### 4.3 Hormonal Imbalance

#### 4.3.1 Estrogen Signaling

Endometriosis is an estrogen‐dependent disorder, with ectopic endometrial tissue expressing estrogen receptors (ERα and ERβ) and progesterone receptors (PR) [90, 91]. Estrogen promotes ectopic tissue growth by stimulating cell proliferation, angiogenesis, and inflammation [92]. In women with endometriosis, the ectopic tissue has increased aromatase activity, leading to local estrogen production [93]. Additionally, ERα expression is increased in ectopic tissue, enhancing estrogen sensitivity [94, 95]. Progesterone resistance is another key feature of endometriosis: PR expression is reduced in ectopic tissue, and progesterone fails to inhibit cell proliferation and promote differentiation [96, 97]. This progesterone resistance contributes to the persistence and growth of ectopic tissue [98]. The estrogen–progesterone imbalance is further amplified by altered metabolism of steroid hormones in the peritoneal microenvironment, creating a feed‐forward loop that sustains ectopic lesion survival and progression.

#### 4.3.2 Other Hormones

Other hormones involved in endometriosis pathogenesis include prostaglandins, growth factors, and insulin‐like growth factors (IGFs) [99]. Prostaglandins, particularly PGE2, are increased in endometriosis, promoting inflammation, angiogenesis, and pain [100]. Growth factors such as vascular endothelial growth factor (VEGF), epidermal growth factor, and platelet‐derived growth factor stimulate angiogenesis and cell proliferation [101]. IGFs, including IGF‐1 and IGF‐2, promote cell growth and survival by activating the PI3K/Akt signaling pathway [102]. These hormones crosstalk with estrogen signaling to synergistically enhance lesion growth, while their dysregulation also contributes to treatment resistance in refractory cases.

### 4.4 Oxidative Stress

Oxidative stress, characterized by an imbalance between reactive oxygen species (ROS) and antioxidant defenses, is a key mechanism in endometriosis [103, 104]. Ectopic endometrial tissue produces increased levels of ROS, which damage DNA, proteins, and lipids, and promote inflammation and cell proliferation [105]. Antioxidant defenses, including superoxide dismutase, catalase, and glutathione peroxidase, are reduced in endometriosis, exacerbating oxidative stress [106, 107]. ROS also activate signaling pathways such as NF‐κB and MAPK, further amplifying the inflammatory response [108]. Environmental factors such as EDCs and cigarette smoking increase ROS production, contributing to disease development [109]. Notably, oxidative stress‐induced DNA damage can drive epigenetic modifications and genomic instability, creating a permissive environment for ectopic tissue invasion. Antioxidant supplementation has shown preliminary efficacy in mitigating symptoms, highlighting therapeutic potential.

### 4.5 Abnormal Cell Invasion and Proliferation

#### 4.5.1 ECM Remodeling

ECM remodeling is critical for the invasion and implantation of ectopic endometrial cells [110, 111]. MMPs, a family of zinc‐dependent endopeptidases, are increased in endometriosis, degrading ECM components such as collagen, fibronectin, and laminin [112]. Key MMPs involved include MMP‐2, MMP‐3, MMP‐9, and MMP‐13 [113]. Tissue inhibitors of metalloproteinases (TIMPs), which regulate MMP activity, are reduced in endometriosis, further promoting ECM degradation [114]. This ECM remodeling allows ectopic endometrial cells to invade surrounding tissues and establish ectopic lesions. Notably, MMP overexpression is tightly regulated by proinflammatory cytokines and estrogen signaling, forming a regulatory loop that amplifies invasive potential. Targeting MMP–TIMP balance has emerged as a promising therapeutic strategy in preclinical models.

#### 4.5.2 Cell Adhesion and Proliferation

Cell adhesion molecules (CAMs), including integrins, cadherins, and selectins, are dysregulated in endometriosis, promoting cell adhesion and invasion [115]. For example, integrin αvβ3 is increased in ectopic tissue, mediating adhesion to the ECM [116]. E‐cadherin, which maintains epithelial cell polarity, is reduced, leading to increased cell motility [117]. Cell proliferation is enhanced in ectopic tissue due to increased expression of cyclins and reduced expression of cyclin‐dependent kinase inhibitors [118, 119]. For example, cyclin D1 is increased, promoting cell cycle progression, while p27Kip1 is reduced, removing a key growth constraint [120]. The dysregulation of CAMs and cell cycle regulators is also linked to epigenetic modifications and oxidative stress, integrating multiple pathogenic pathways to drive ectopic tissue expansion.

**FIGURE 3:**
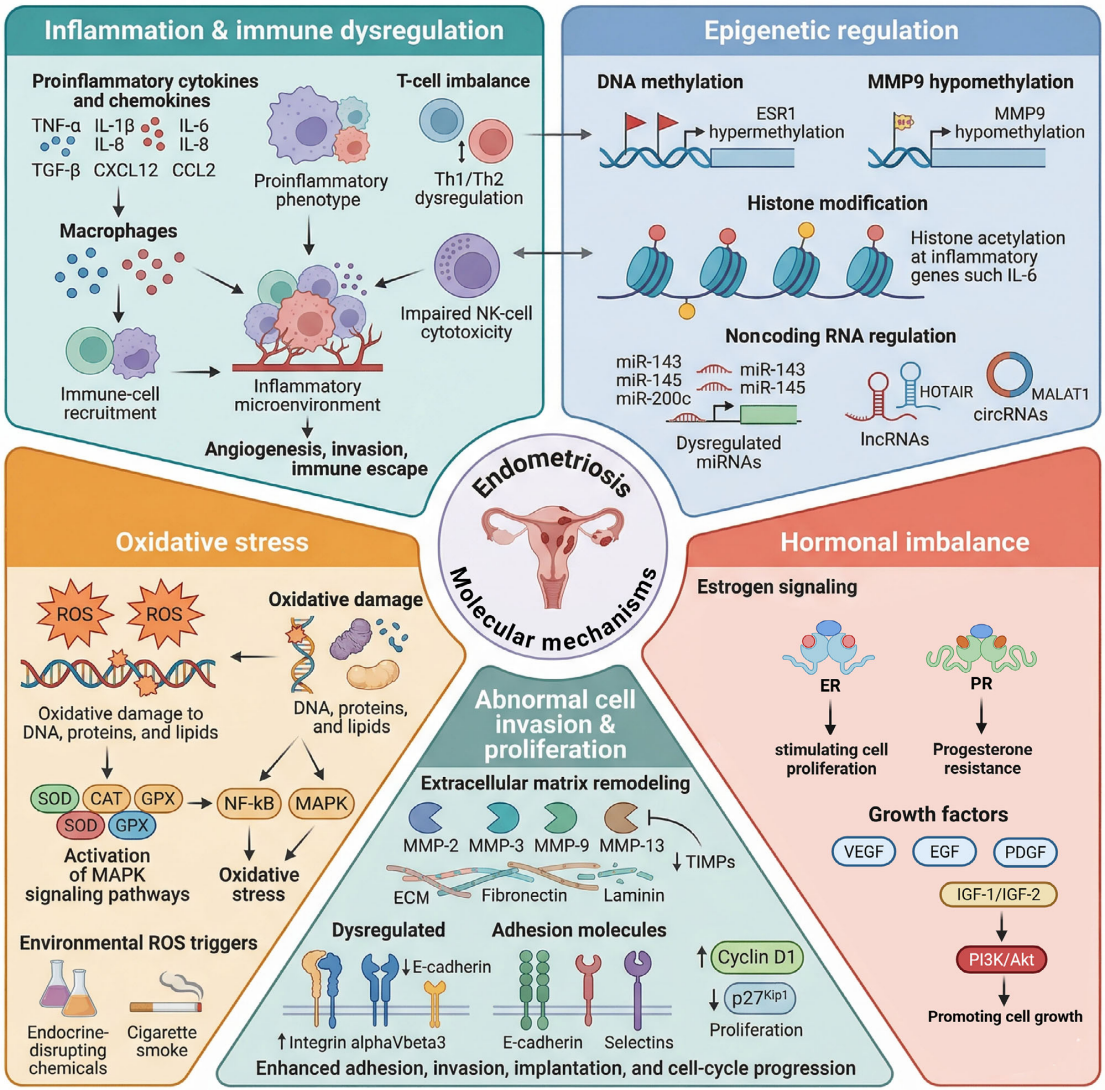
Core molecular mechanisms underlying endometriosis pathogenesis. This diagram systematically presents the five core and mutually interactive molecular pathways that drive the initiation and progression of endometriosis. Inflammation and immune dysregulation, characterized by overactivated macrophages, elevated proinflammatory cytokines, and impaired NK cell cytotoxicity, initiate a chronic proinflammatory microenvironment. Epigenetic abnormalities, including DNA methylation, histone modification, and dysregulated microRNAs, alter gene expression related to hormone sensitivity and cell invasion. Estrogen dominance and progesterone resistance form the hormonal basis for continuous lesion growth. Oxidative stress enhances DNA damage and inflammatory signaling, while excessive extracellular matrix degradation and abnormal cell adhesion and proliferation enable ectopic implantation. These pathways do not function independently but crosstalk intensively to construct a self‐reinforcing pathological network. This figure was created with BioRender.com.

The molecular pathogenesis of endometriosis relies on intensive crosstalk among all aforementioned pathways: chronic inflammation triggers epigenetic alterations, which further dysregulate hormone receptor expression; oxidative stress amplifies inflammation and promotes cell invasion; and hormonal imbalance sustains a prosurvival microenvironment. These pathways form a self‐reinforcing regulatory network that collectively drives the initiation, implantation, and progression of ectopic lesions (Figure 3).

## 5 Diagnosis of Endometriosis

Section 5 systematically reviews the diagnostic workflow of endometriosis, from traditional clinical and imaging methods to laparoscopic gold standard and emerging noninvasive biomarkers, highlighting strengths, limitations, and future directions for early and accurate diagnosis.

### 5.1 Traditional Diagnostic Methods

#### 5.1.1 Clinical History and Physical Examination

Clinical history and physical examination are the first steps in the diagnosis of endometriosis. Women with symptoms such as CPP, dysmenorrhea, dyspareunia, or infertility should be evaluated for endometriosis [121, 122]. Physical examination includes a pelvic exam to assess for tender nodules in the posterior cul‐de‐sac, adnexal masses, or uterine enlargement [123]. However, physical examination has limited sensitivity and specificity, particularly for early‐stage endometriosis or deep infiltrating endometriosis (DIE) [124]. Moreover, many patients experience long delays in diagnosis due to nonspecific symptoms and low clinical suspicion, especially in adolescent and young adult populations.

#### 5.1.2 Imaging Modalities

Imaging modalities are important for supporting the diagnosis of endometriosis [125, 126]. Transvaginal ultrasound (TVUS) is the first‐line imaging test, with a sensitivity of 70–90% and specificity of 80–95% for ovarian endometriomas [127]. TVUS can also detect DIE lesions, particularly those involving the rectovaginal septum or bladder. Magnetic resonance imaging (MRI) is more sensitive and specific for DIE, with a sensitivity of 85–95% and specificity of 90–98% [128]. MRI is particularly useful for preoperative planning, as it can accurately assess the extent of lesions and involvement of adjacent organs [129]. Computed tomography is not recommended for routine diagnosis, as it has lower sensitivity and specificity than TVUS and MRI [130]. Operator experience significantly influences TVUS accuracy, while MRI remains more reliable for evaluating complex pelvic anatomy and multifocal disease.

#### 5.1.3 Laparoscopy

Laparoscopy is the gold standard for the diagnosis of endometriosis, allowing direct visualization of ectopic lesions [131]. Laparoscopy can confirm the presence of endometriosis, assess the stage of disease (using the revised American Society for Reproductive Medicine classification), and perform therapeutic interventions [132]. However, laparoscopy is an invasive procedure with associated risks, including infection, bleeding, and organ damage [133, 134]. Additionally, laparoscopy has limited sensitivity for early‐stage endometriosis, as small lesions may be missed [135]. Histopathological confirmation is often required to confirm the diagnosis, as visual inspection alone may lead to false‐positive or false‐negative results.

### 5.2 Novel Noninvasive Biomarkers

#### 5.2.1 Serum Biomarkers

Serum biomarkers are promising noninvasive tools for the diagnosis of endometriosis [136, 137]. CA‐125 is the most well‐studied serum biomarker, but it has limited sensitivity (40–60%) and specificity (70–80%) for endometriosis, as it is also elevated in other conditions such as ovarian cancer and pregnancy [138, 139]. Novel serum biomarkers include CA‐199, HE4, and miRNAs [140]. For example, serum levels of miR‐125b, miR‐155, and miR‐200c are dysregulated in women with endometriosis, with a combined sensitivity of 85% and specificity of 90% [141]. Other serum biomarkers include inflammatory cytokines (e.g., TNF‐α, IL‐6) and growth factors (e.g., VEGF), but their clinical utility is limited by variability [142]. Combined panels of multiple serum markers rather than single markers are expected to overcome low specificity and provide more reliable screening for early‐stage disease.

#### 5.2.2 Urine and Endometrial Biomarkers

Urine biomarkers are emerging as noninvasive alternatives to serum biomarkers [143, 144]. Urinary levels of MMP‐9, VEGF, and miRNAs have been shown to be elevated in women with endometriosis [145]. For example, urinary miR‐143 levels have a sensitivity of 75% and specificity of 80% for endometriosis [145]. Endometrial biomarkers, including miRNAs, lncRNAs, and proteins, are also being investigated. For example, endometrial expression of miR‐199a‐5p and miR‐200a is reduced in women with endometriosis, with a combined sensitivity of 88% and specificity of 92% [146]. Endometrial biopsy is a minimally invasive procedure, making it a promising tool for biomarker‐based diagnosis. Urine sampling is completely noninvasive and easily repeatable, while endometrial biomarkers offer higher tissue specificity, both representing valuable directions for next‐generation diagnostic development.

### 5.3 Diagnostic Algorithms and Future Directions

The diagnosis of endometriosis remains challenging, and there is no single diagnostic test with sufficient sensitivity and specificity. Current diagnostic algorithms combine clinical history, physical examination, imaging, and, in some cases, laparoscopy [130, 147]. For women with typical symptoms, TVUS is recommended as the first‐line imaging test; if TVUS is inconclusive, MRI may be performed [123, 148]. Laparoscopy is reserved for cases where the diagnosis is uncertain or when therapeutic intervention is planned [131]. Delayed diagnosis often leads to disease progression and reduced quality of life, highlighting the urgent need for faster and more accurate screening tools. Future directions include the development of multibiomarker panels, combining serum, urine, and endometrial biomarkers, to improve diagnostic accuracy [149]. Additionally, artificial intelligence (AI) and machine learning algorithms are being used to analyze imaging and biomarker data, potentially enhancing diagnostic efficiency [150, 151].

**FIGURE 4:**
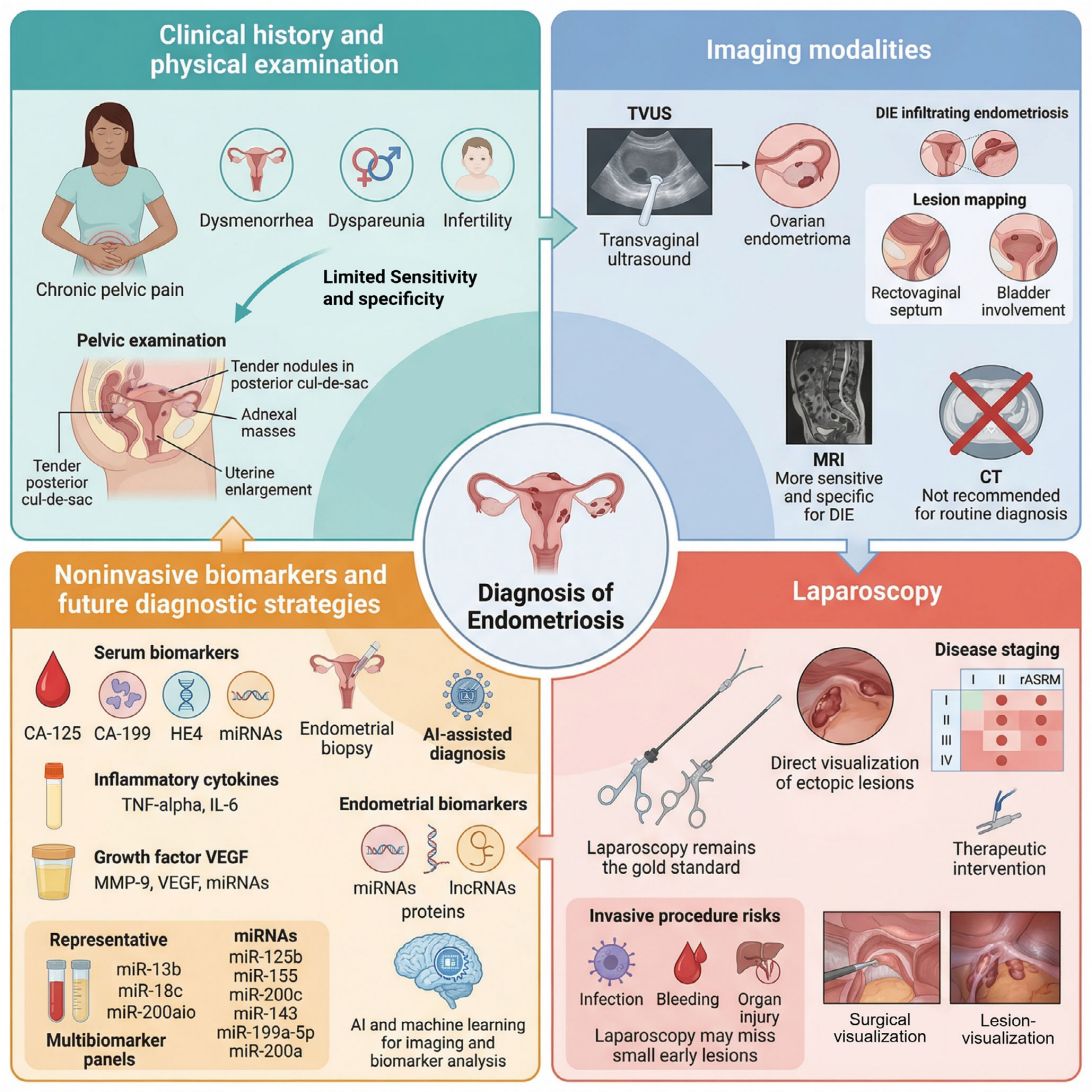
Diagnostic workflow and modalities for endometriosis. This figure presents a standardized, stepwise diagnostic workflow for endometriosis that integrates clinical evaluation, imaging, invasive confirmation, and novel noninvasive detection. It begins with clinical history collection and pelvic examination, followed by first‐line transvaginal ultrasound (TVUS) and complementary magnetic resonance imaging (MRI) for evaluating deep infiltrating lesions. Laparoscopy, as the gold standard, enables direct visualization and histological verification. Emerging noninvasive biomarkers including serum microRNAs, CA‐125, urinary proteins, and endometrial molecular markers are highlighted as promising tools for early screening. The diagram emphasizes a layered diagnostic strategy to improve accuracy, reduce delay, and enable early identification, especially in adolescent and young adult populations. This figure was created with BioRender.com.

The diagnosis of endometriosis relies on a combination of clinical history, physical examination, imaging, and laparoscopy (Figure 4). Traditional diagnostic methods have limitations, particularly for early‐stage disease. Moreover, inconsistent presentation and atypical symptoms further complicate timely identification in young and adolescent populations. Novel noninvasive biomarkers, including serum, urine, and endometrial biomarkers, show promise for improving diagnostic accuracy. Future research should focus on developing multibiomarker panels and AI‐based diagnostic tools to enable early, noninvasive diagnosis of endometriosis.

## 6 Management of Endometriosis

Section 6 presents a stratified, patient‐centered management algorithm for endometriosis, covering first‐line hormonal therapies, minimally invasive surgery, emerging targeted treatments, and fertility preservation strategies tailored to symptoms, disease severity, and reproductive goals.

### 6.1 Hormonal Therapies

#### 6.1.1 Oral Contraceptives

Oral contraceptives (OCs) are the first‐line hormonal therapy for endometriosis, particularly for women with dysmenorrhea and CPP [152]. OCs contain estrogen and progestin, which suppress ovulation, reduce estrogen levels, and thin the endometrial lining [153]. Continuous use of OCs (without a placebo week) is more effective than cyclic use for pain relief [154]. The side effects of OCs include nausea, breast tenderness, and irregular bleeding, but these are generally mild [155]. OCs are not recommended for women with a history of thromboembolic disease, breast cancer, or liver disease [156].

#### 6.1.2 Progestins

Progestins are another important class of hormonal therapies for endometriosis [154, 157]. They work by suppressing estrogen receptor expression, inhibiting cell proliferation, and promoting apoptosis [91]. Common progestins used include medroxyprogesterone acetate (MPA), norethindrone acetate, and dienogest [158]. Dienogest is a highly selective progestin with potent antiendometriosis effects, reducing pain and lesion size in 70–80% of women [159]. The side effects of progestins include weight gain, mood changes, and irregular bleeding [154, 160]. Progestins are contraindicated in women with progesterone‐dependent tumors or liver disease.

#### 6.1.3 Gonadotropin‐Releasing Hormone Agonists and Antagonists

Gonadotropin‐releasing hormone (GnRH) agonists and antagonists are used for the treatment of severe endometriosis or when other hormonal therapies are ineffective [157, 161]. GnRH agonists (e.g., leuprolide, goserelin) suppress gonadotropin secretion, leading to hypoestrogenism and regression of ectopic lesions [162]. GnRH antagonists (e.g., elagolix, ganirelix) have a similar mechanism of action but do not cause an initial flare of symptoms [163]. The side effects of GnRH agonists and antagonists include hot flashes, vaginal dryness, and bone loss, which can be mitigated with add‐back therapy (estrogen‐progestin replacement) [164]. These therapies are not recommended for long‐term use (more than 6 months) due to the risk of bone loss [165].

### 6.2 Surgical Interventions

#### 6.2.1 Laparoscopic Surgery

Laparoscopic surgery is the preferred surgical approach for endometriosis, as it is minimally invasive with shorter recovery time and fewer complications [166, 167]. Surgical procedures include excision of ectopic lesions, cystectomy for ovarian endometriomas, and lysis of adhesions [166]. Excision of lesions is more effective than ablation (destruction of lesions) for pain relief and reducing recurrence [168]. For women with infertility, laparoscopic surgery can improve pregnancy rates, particularly for mild‐to‐moderate endometriosis [169]. The recurrence rate after laparoscopic surgery is approximately 20–30% within 5 years [170]. Furthermore, laparoscopy allows for precise visualization and complete removal of deep infiltrating lesions and peritoneal implants, which helps reduce CPP and improve postoperative quality of life. Skillful dissection and meticulous hemostasis during surgery can further reduce adhesion formation and lower the risk of postoperative complications. Individualized surgical planning based on disease severity and patient age also contributes to better long‐term outcomes.

#### 6.2.2 Laparotomy and Hysterectomy

Laparotomy is reserved for severe cases of endometriosis, such as extensive DIE or large adnexal masses, where laparoscopic surgery is not feasible [171]. Hysterectomy, with or without oophorectomy, is considered for women with severe symptoms who do not wish to preserve fertility [172]. Hysterectomy with bilateral oophorectomy is the most effective surgical procedure for eliminating endometriosis‐related pain, but it induces menopause and may have long‐term health consequences [173]. Oophorectomy should be avoided in women of reproductive age unless necessary, as it reduces bone density and increases the risk of cardiovascular disease [174]. In addition, laparotomy is associated with longer hospital stays, higher infection rates, and slower postoperative recovery compared with laparoscopy. For patients undergoing hysterectomy, preoperative assessment of ovarian reserve and cardiovascular health is essential to balance symptom control and long‐term safety. Individualized decisions regarding oophorectomy should account for age, menopausal status, family history, and patient preferences to minimize metabolic and bone health risks.

### 6.3 Emerging Targeted Therapies

#### 6.3.1 Anti‐Inflammatory and Immunotherapeutic Agents

Anti‐inflammatory agents, including nonsteroidal anti‐inflammatory drugs (NSAIDs) and biologic therapies, are being investigated for the treatment of endometriosis [175]. NSAIDs are used for pain relief but have no effect on lesion growth [176]. Biologic therapies targeting cytokines such as TNF‐α (e.g., infliximab, adalimumab) and IL‐6 (e.g., tocilizumab) have shown promise in preclinical studies and small clinical trials [177]. For example, infliximab reduced pain and lesion size in women with severe endometriosis [178]. Immunotherapeutic agents, such as checkpoint inhibitors, are also being explored, as endometriosis is associated with immune dysregulation [179]. These agents can restore local immune surveillance, suppress chronic inflammatory signaling, and inhibit the proliferation and invasion of ectopic lesions. Combined use with hormonal therapies may further enhance clinical efficacy and reduce recurrence rates in refractory cases.

#### 6.3.2 Epigenetic and Molecular Targeted Therapies

Epigenetic targeted therapies, including DNA methyltransferase inhibitors and histone deacetylase (HDAC) inhibitors, are being investigated for the treatment of endometriosis [180]. For example, the HDAC inhibitor vorinostat reduced lesion size and inflammation in a murine model of endometriosis [181]. Molecular targeted therapies include inhibitors of MMPs, VEGF, and the PI3K/Akt/mTOR signaling pathway [115, 182]. For example, the MMP inhibitor marimastat reduced lesion growth in preclinical studies [183], and the VEGF inhibitor bevacizumab improved pain and reduced lesion size in a small clinical trial [184]. These targeted therapies have the potential to improve treatment efficacy and reduce side effects compared with traditional hormonal therapies. Moreover, they can regulate abnormal gene expression profiles, reverse pathological epigenetic modifications, and block key signaling pathways that drive lesion progression. Their long‐term safety and optimal combination strategies require further evaluation in large‐scale, multicenter clinical trials.

### 6.4 Fertility Preservation and Management

#### 6.4.1 Fertility Preservation Options

Women with endometriosis who wish to preserve fertility have several options, including ovarian tissue cryopreservation, oocyte cryopreservation, and embryo cryopreservation [185, 186, 187]. Ovarian tissue cryopreservation is the only option for pre‐pubertal girls or women who cannot undergo ovarian stimulation [188]. Oocyte cryopreservation is a viable option for women of reproductive age, with pregnancy rates similar to those of fresh oocytes [189]. Embryo cryopreservation is the most effective fertility preservation option, but it requires a partner or donor sperm [190]. Each method carries distinct ethical, clinical, and timing considerations; shared decision‐making between patients and multidisciplinary teams is essential to select the optimal strategy. Individualized plans should account for disease severity, age, treatment urgency, and personal reproductive values to maximize future fertility chances.

#### 6.4.2 Fertility Management Strategies

Fertility management for women with endometriosis includes lifestyle modifications, medical therapy, and assisted reproductive technology (ART) [191]. Lifestyle modifications such as weight loss, exercise, and vitamin D supplementation may improve fertility [192]. Medical therapy with clomiphene citrate or letrozole may be used for ovulation induction in women with mild endometriosis [193]. ART, including in vitro fertilization (IVF), is recommended for women with moderate‐to‐severe endometriosis or unexplained infertility [194]. IVF success rates are lower in women with endometriosis, but pre‐IVF surgical excision of lesions may improve outcomes [195]. Additionally, early intervention and regular monitoring can significantly enhance reproductive outcomes, while psychological support and stress reduction strategies further support reproductive health and treatment adherence.

**FIGURE 5:**
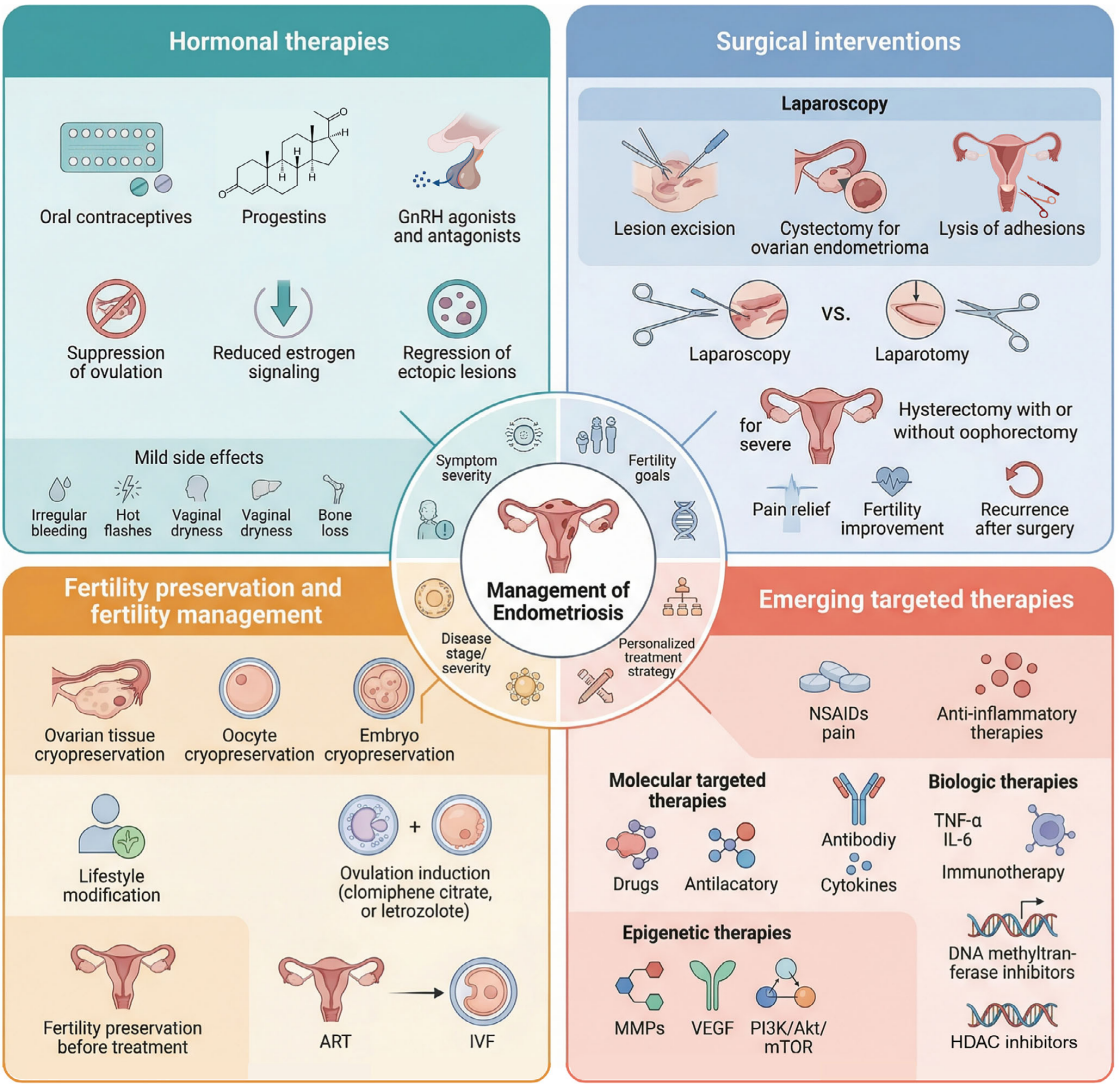
Clinical management algorithm for endometriosis. This flowchart provides a patient‐centered, stratified clinical management algorithm for endometriosis according to disease severity, symptoms, and fertility requirements. First‐line treatments include combined oral contraceptives and progestins for pain relief. GnRH agonists and antagonists are recommended for moderate to severe cases. Minimally invasive laparoscopic surgery is indicated for drug‐resistant pain, large endometriomas, deep lesions, or infertility‐related needs. For patients pursuing pregnancy, fertility preservation techniques including oocyte/embryo cryopreservation and assisted reproductive technology are integrated. Emerging targeted therapies such as anti‐inflammatory agents, epigenetic modulators, and antiangiogenic drugs are also included as future directions. The algorithm emphasizes personalized decision‐making and long‐term follow‐up to reduce recurrence and improve quality of life. This figure was created with BioRender.com.

The management of endometriosis is tailored to the individual patient's symptoms, fertility goals, and disease severity. Hormonal therapies are first‐line for pain relief, while surgical interventions are used for severe disease or infertility. Emerging targeted therapies, including anti‐inflammatory, immunotherapeutic, and epigenetic agents, show promise for improving treatment efficacy. Fertility preservation and management are important considerations for women of reproductive age (Figure 5). Future research should focus on developing personalized treatment strategies based on disease subtype and molecular profile. Long‐term follow‐up is essential to monitor disease recurrence, chronic pain, and comorbid conditions, while multidisciplinary care involving gynecologists, reproductive specialists, and nurses ensures comprehensive, patient‐centered care throughout the treatment journey.

## 7 Preclinical Models and Clinical Trials

Relevant preclinical animal experiments and clinical trials for endometriosis are being extensively conducted worldwide, with preclinical studies focusing on pathogenesis exploration and novel target identification, and clinical trials expanding rapidly with a focus on hormonal interventions and emerging nonhormonal therapies, presenting distinct regional and research phase characteristics. Multicenter collaborations and standardized protocols are increasingly emphasized to improve data comparability and accelerate translation. Both mechanistic studies and patient‐centered outcomes are being integrated to support more effective and individualized treatment strategies.

### 7.1 Preclinical Animal Models

#### 7.1.1 Rodent Models

Rodent models are the most commonly used preclinical models for endometriosis research [196]. The most widely used model is the murine model of endometriosis, which involves the transplantation of endometrial tissue into the peritoneal cavity of immunocompetent or immunodeficient mice [197]. This model recapitulates key features of human endometriosis, including ectopic lesion growth, inflammation, and pain [198]. Rat models are also used, with similar characteristics to murine models [199]. Other rodent models include the induced endometriosis model (using EDCs or hormonal manipulation) and the genetic model (using knockout mice) [200]. These models are useful for studying disease pathogenesis and testing novel therapies.

#### 7.1.2 Nonrodent Models

Nonrodent models, including primate and rabbit models, are less commonly used but provide a more translational approach. Primate models, such as rhesus macaques, develop spontaneous endometriosis, making them ideal for studying natural disease progression [201]. Rabbit models involve the transplantation of endometrial tissue into the peritoneal cavity, with lesions similar to those in humans [199]. Nonrodent models are more expensive and time‐consuming than rodent models but are valuable for testing therapies prior to clinical trials.

### 7.2 Translation From Preclinical Models to Clinical Trials

The translation of preclinical findings to clinical trials is a critical step in the development of new therapies for endometriosis. However, there are several challenges: rodent models may not fully recapitulate human endometriosis, and preclinical studies often use high doses of drugs that may not be safe in humans. To address these challenges, researchers are using more translational models, such as primate models, and conducting mechanistic studies to understand the molecular basis of drug efficacy. Additionally, clinical trials are increasingly incorporating biomarkers to select patients who are most likely to respond to treatment.

Preclinical mechanistic research has uncovered multiple potential therapeutic targets for endometriosis through cellular and animal model studies, laying a foundation for subsequent clinical translation. A key finding revealed that ectopic endometrial mesenchymal stromal cells can activate adjacent ovarian stromal cells via the WNT5A pathway, which in turn triggers abnormal cellular proliferation and inflammatory responses in lesions [202, 203]. This discovery identifies WNT5A as a promising target for developing novel diagnostic tools and targeted therapies. Additionally, leveraging the AI‐based bioinformatics platform PandaOmics, researchers screened out GBP2 and HCK as candidate therapeutic targets, establishing a direct link between immune regulatory pathways and endometriosis onset and progression [204]. This finding opens up new directions for developing immune‐targeted interventions, which are expected to inhibit ectopic lesion growth and improve clinical outcomes by regulating immune cell function and inflammatory signaling. These preclinical results provide critical theoretical support for the design of subsequent Phase I/II clinical trials and the development of innovative drugs.

### 7.3 Clinical Trials

As of April 7, 2025, a total of 744 interventional pharmaceutical clinical trials for endometriosis were included in the global analysis (excluding observational studies), with the number of trials rising steadily‐especially early‐phase (I/II) trials surging, reflecting a boom in innovative drug development [205]. Geographically, China and the US dominate global research, with Japan, Italy, and Germany as secondary contributors, while the Asia‐Pacific, Latin America, and Africa still have great potential for development. Trials are mainly sponsored by the pharmaceutical industry, with academic institutions driving cutting‐edge basic research, and the need for closer industry–academia cooperation is highlighted.

Hormonal therapies, particularly GnRH antagonists, remain the mainstream of clinical trials, with several key ongoing and completed studies yielding important findings (see Table 1) [206]. Nonhormonal therapies, represented by P2×3 receptor antagonists, were once considered promising for pain management, but recent clinical trials showed limited efficacy: eliapixant (NCT04614246, Phase IIb) and gefapixant (NCT03654326, proof‐of‐concept) both failed to show statistically or clinically significant pain relief compared with placebo, reflecting the complexity of endometriosis‐associated pain regulation [207, 208]. A small percentage of clinical trials were terminated early, indicating the need for improved risk management, efficacy prediction, and biomarker development to reduce trial failure rates.

Preclinical animal models, particularly rodent models, are valuable for studying endometriosis pathogenesis and testing novel therapies. Ongoing clinical trials are investigating targeted therapies and diagnostic biomarkers, with promising preliminary findings. The translation of preclinical findings to clinical trials is challenging but essential for improving patient outcomes. Future research should focus on developing more translational models and incorporating biomarkers into clinical trials to enable personalized medicine.

In summary, current clinical trials are dominated by GnRH antagonists, but their long‐term use is limited by bone density loss; nonhormonal therapies are still in the exploratory stage with unmet needs. Preclinical research has identified multiple novel targets, and their clinical translation is the key direction for future endometriosis research. (Table 2)

**TABLE 2:** Ongoing and completed clinical trials of pharmacotherapies for endometriosis.

Pathway/mechanism	Drug	NCT no./trial name	Phase	Status	Core objectives	Preliminary findings
GnRH receptor antagonism [206]	Elagolix + ABT	—	III	Ongoing (48 months)	Evaluate long‐term efficacy, safety, and bone density for moderate‐severe pain	12‐month data: improved dysmenorrhea/pelvic pain/fatigue, but higher BMD loss vs. placebo
GnRH receptor antagonism [206]	Relugolix combination therapy	SPIRIT 1/2 (open‐label extension)	—	Completed	Assess 2‐year efficacy/safety for pain relief	Sustained pain relief over 104 weeks; initial mild BMD loss stabilized with continued treatment
GnRH receptor antagonism [206]	Linzagolix	EDELWEISS 3	III	Completed	Compare monotherapy/combination therapy vs. placebo for pain relief	200 mg + ABT alleviated two pain types; 75 mg monotherapy only reduced dysmenorrhea
P2×3 receptor antagonism [207]	Eliapixant	NCT04614246	IIb	Completed	Evaluate efficacy/safety for pelvic pain	No significant pain relief vs. placebo
P2×3 receptor antagonism [208]	Gefapixant (45 mg bid)	NCT03654326	Proof‐of‐concept	Completed	Assess superior pain relief vs. placebo	No better efficacy than placebo
Unspecified [208]	HMI‐115	CTR20255175	III	Ongoing	Evaluate 24‐week efficacy/safety of HMI‐115 for moderate‐severe pain (28‐week extension)	No preliminary findings available

Abbreviations: BMD, bone mineral density; GnRH, gonadotropin‐releasing hormone

## 8 Conclusion and Prospects

### 8.1 Summary of Key Findings

Endometriosis is a complex, chronic gynecological disorder with a high prevalence and significant clinical impact. This review has provided a comprehensive overview of endometriosis, covering its epidemiology, risk factors, molecular mechanisms, diagnosis, and management. Key findings include: (1) endometriosis is a globally prevalent disorder with significant regional and demographic variations; (2) genetic, environmental, and lifestyle factors contribute to disease risk; (3) disease pathogenesis involves inflammation, epigenetic regulation, hormonal imbalance, oxidative stress, and abnormal cell invasion; (4) diagnosis relies on a combination of clinical history, imaging, and laparoscopy, with novel biomarkers showing promise; (5) management includes hormonal therapies, surgical interventions, and emerging targeted therapies; and (6) preclinical models and ongoing clinical trials are bridging basic research and clinical translation. Moreover, emerging evidence underscores the critical role of gut microbiota, EDCs, and chronic low‐grade inflammation in driving disease progression and treatment resistance. Additionally, long‐term comorbidities such as chronic pain, infertility, and psychological distress substantially reduce quality of life and highlight the urgent need for more effective, personalized, and mechanism‐based therapeutic strategies.

### 8.2 Current Challenges and Unresolved Questions

Despite significant progress, several challenges and unresolved questions remain. First, the exact mechanism of endometrial tissue dissemination and implantation is not fully understood, and the role of the microbiome in disease development requires further investigation. Second, noninvasive diagnostic biomarkers with sufficient sensitivity and specificity are lacking, leading to delayed diagnosis. Third, existing treatments are primarily symptomatic, with high recurrence rates, and there is a need for disease‐modifying therapies. Fourth, the heterogeneity of endometriosis makes personalized treatment challenging, and the identification of disease subtypes is needed. Fifth, the long‐term comorbidities of endometriosis, such as ovarian cancer and cardiovascular disease, require further study. In addition, the complex interactions between hormonal dysregulation, immune evasion, metabolic stress, and epigenetic modifications in disease progression remain largely unexplored. Furthermore, the lack of standardized preclinical models and large‐scale longitudinal clinical cohorts limits the validation of novel biomarkers and therapeutic targets. There is also an urgent need to address disparities in disease recognition, access to specialized care, and patient‐centered outcomes across different populations. Finally, the integration of multiomics data and AI‐driven algorithms will be critical to uncover novel pathogenic mechanisms and translate scientific discoveries into clinically actionable strategies for endometriosis.

### 8.3 Future Directions and Research Priorities

Future research should focus on addressing these challenges and advancing our understanding of endometriosis. Key research priorities include: (1) investigating the role of the microbiome and environmental factors in disease pathogenesis; (2) developing multibiomarker panels and AI‐based diagnostic tools for early, noninvasive diagnosis; (3) identifying novel therapeutic targets and developing disease‐modifying therapies; (4) subclassifying endometriosis based on molecular profiles to enable personalized treatment; (5) conducting long‐term studies to understand the comorbidities of endometriosis; and (6) improving the translation of preclinical findings to clinical trials. Moreover, future investigations should explore the crosstalk between hormonal signaling, immune dysregulation, and metabolic dysfunction in driving disease progression, as well as the long‐term impacts of current treatments on quality of life, mental health, and chronic disease risk. Additionally, there is a need for increased collaboration between basic researchers, clinicians, and patient advocates to accelerate research and improve patient outcomes. Multidisciplinary teams integrating gynecology, immunology, genetics, microbiology, and data science will be essential to uncover disease mechanisms, validate biomarkers, and design curative interventions rather than symptomatic management alone.

### 8.4 Clinical Implications and Future of Endometriosis Management

The future of endometriosis management lies in precision medicine, with treatments tailored to the individual patient's disease subtype, molecular profile, and fertility goals. Emerging technologies such as single‐cell RNA sequencing and spatial transcriptomics will enable the identification of disease subtypes and novel therapeutic targets. Noninvasive diagnostic biomarkers will allow for early diagnosis and monitoring of disease progression. Targeted therapies, including anti‐inflammatory, immunotherapeutic, and epigenetic agents, will provide more effective and safer treatment options. Additionally, fertility preservation and management will continue to be a key focus, with advances in ART and ovarian tissue cryopreservation improving outcomes for women with endometriosis. Moreover, the integration of multiomics data and AI will further refine risk stratification and enable real‐time monitoring of treatment responses. Long‐term comorbidities such as cardiovascular disease, ovarian cancer risk, and mental health disorders should also be incorporated into routine clinical care to achieve holistic and lifelong management.

Endometriosis is a complex disorder with significant clinical impact, but recent advances in epidemiology, molecular biology, diagnosis, and treatment have improved our understanding and management of the disease. Current challenges include delayed diagnosis, suboptimal treatment efficacy, and disease heterogeneity. Future research should focus on developing noninvasive diagnostic tools, disease‐modifying therapies, and personalized treatment strategies. Translational research must bridge preclinical findings and clinical trials, with standardized protocols and large multicenter cohorts to enhance reproducibility. Greater public awareness and multidisciplinary collaboration among gynecologists, immunologists, geneticists, and microbiologists are also critical to accelerate progress. With continued research and collaboration, we can improve the lives of women with endometriosis and move toward a future where this disorder is effectively diagnosed and managed.

## Author Contributions

All authors made substantial contributions to the conception and design of the review. Kun Wang, Dongyun He, and Yang Wang drafted the manuscript. Xiaojun Liu and Li Liu revised the manuscript for important intellectual content. All authors read and approved the final manuscript.

## Conflicts of Interest

The authors declare no conflicts of interest.

## Ethics Statement

The authors have nothing to report.

## Funding Information

This article was supported by Special Project for Health Research Talents in Jilin Province (2024SCZ76).

## Data Availability

The authors have nothing to report.
